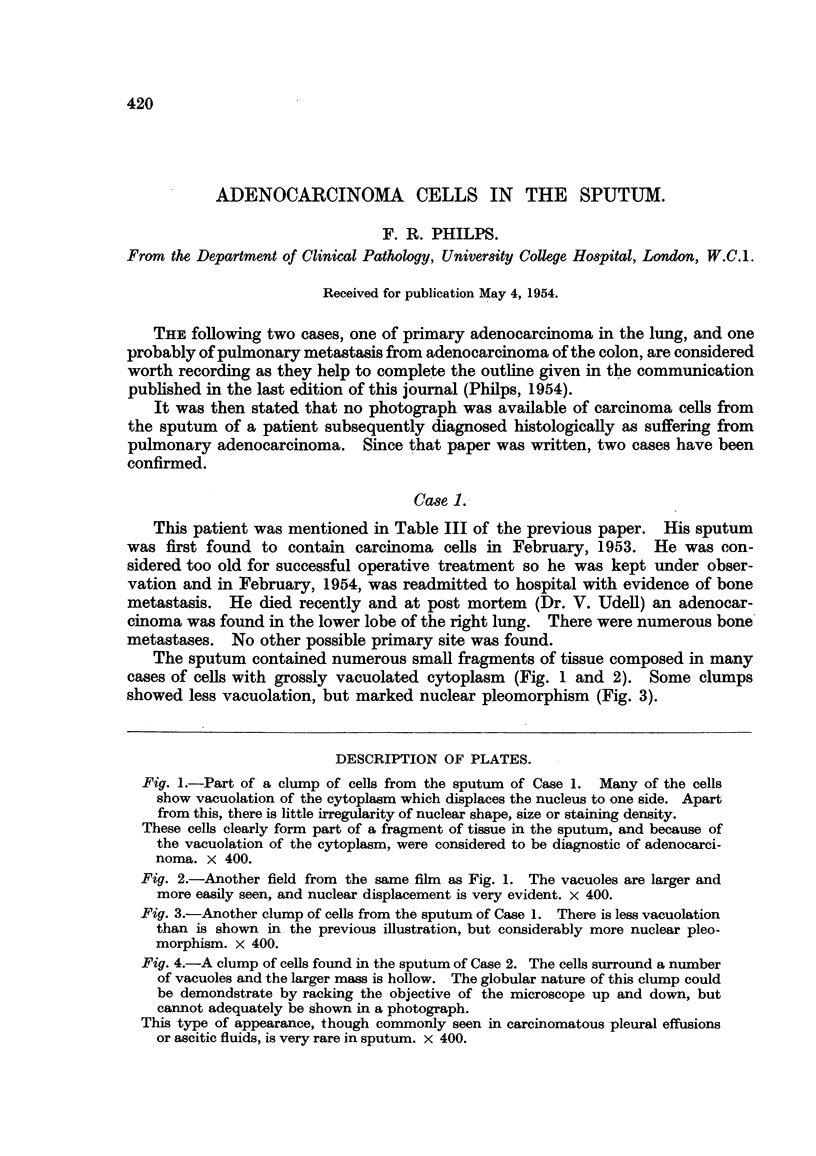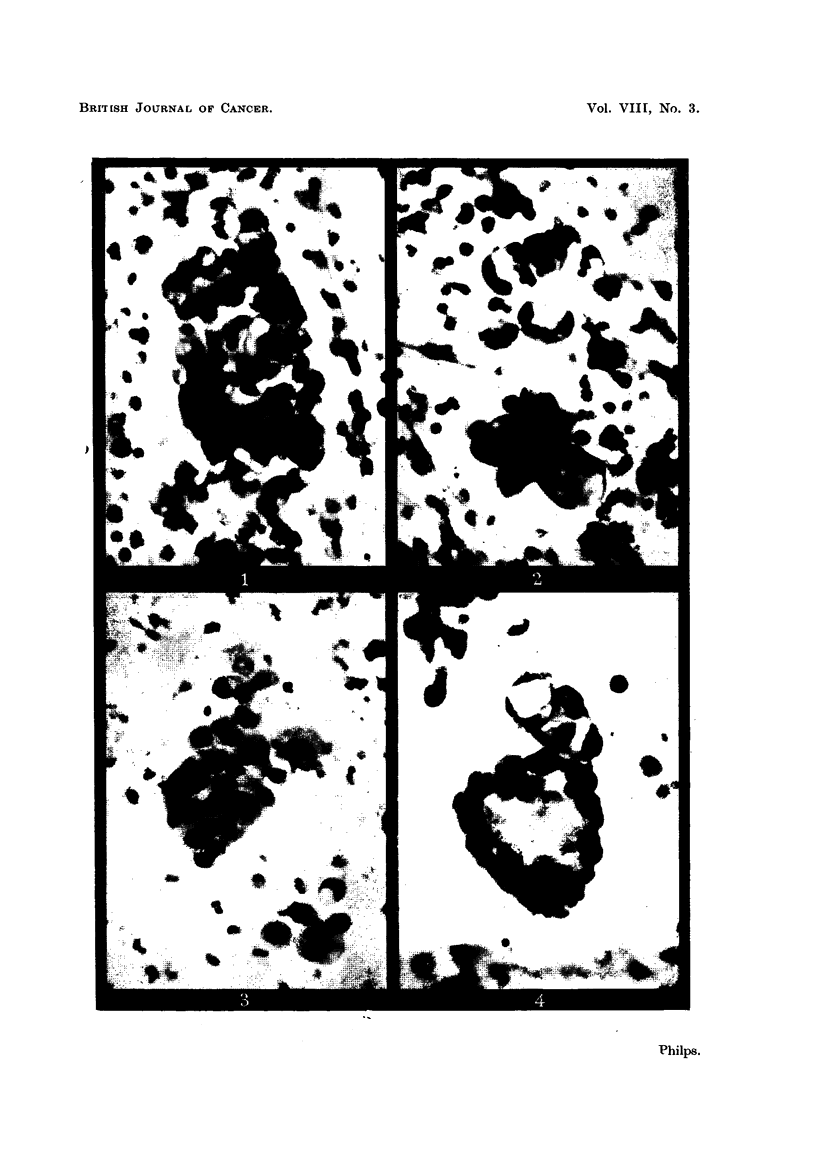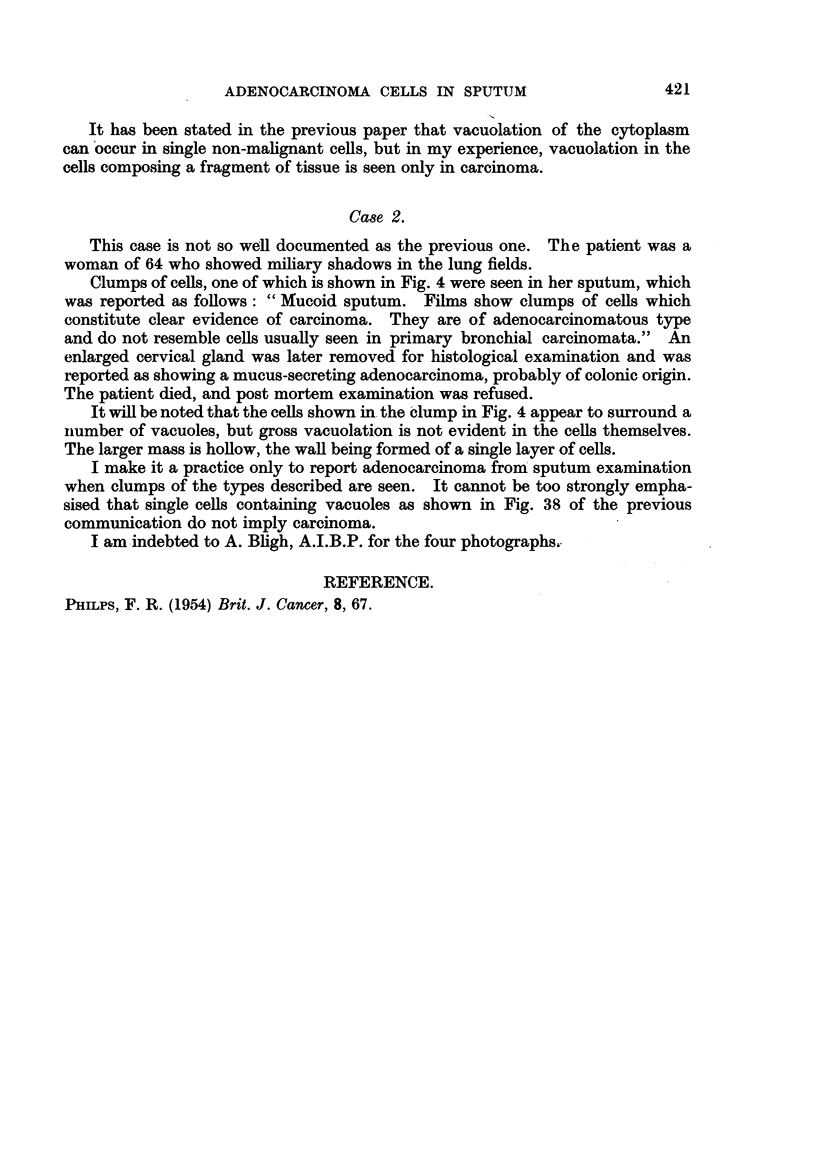# Adenocarcinoma Cells in the Sputum

**DOI:** 10.1038/bjc.1954.43

**Published:** 1954-09

**Authors:** F. R. Philps

## Abstract

**Images:**


### ADENOCARCINOMA CELLS IN THE SPUTUM.

F. R. PHILPS.

*From the Department of Clinical Pathology, University College Hospital, London, W.C.*1.

Received for publication May 4, 1954.

THE following two cases, one of primary adenocarcinoma in the lung, and one probably of pulmonary metastasis from adenocarcinoma of the colon, are considered worth recording as they help to complete the outline given in the communication published in the last edition of this journal (Philps, 1954).

It was then stated that no photograph was available of carcinoma cells from the sputum of a patient subsequently diagnosed histologically as suffering from pulmonary adenocarcinoma. Since that paper was written, two cases have been confirmed.

### *Case 1.*

This patient was mentioned in Table III of the previous paper. His sputum was first found to contain carcinoma cells in February, 1953. He was considered too old for successful operative treatment so he was kept under observation and in February, 1954, was readmitted to hospital with evidence of bone metastasis. He died recently and at post mortem (Dr. V. Udell) an adenocarcinoma was found in the lower lobe of the right lung. There were numerous bone metastases. No other possible primary site was found.

The sputum contained numerous small fragments of tissue composed in many cases of cells with grossly vacuolated cytoplasm (Fig. 1 and 2). Some clumps showed less vacuolation, but marked nuclear pleomorphism (Fig. 3).

---

DESCRIPTION OF PLATES.

*Fig.* 1.—Part of a clump of cells from the sputum of Case 1. Many of the cells show vacuolation of the cytoplasm which displaces the nucleus to one side. Apart from this, there is little irregularity of nuclear shape, size or staining density.

These cells clearly form part of a fragment of tissue in the sputum, and because of the vacuolation of the cytoplasm, were considered to be diagnostic of adenocarcinoma. × 400.

*Fig.* 2.—Another field from the same film as Fig. 1. The vacuoles are larger and more easily seen, and nuclear displacement is very evident. × 400.

*Fig.* 3.—Another clump of cells from the sputum of Case 1. There is less vacuolation than is shown in the previous illustration, but considerably more nuclear pleomorphism. × 400.

*Fig.* 4.—A clump of cells found in the sputum of Case 2. The cells surround a number of vacuoles and the larger mass is hollow. The globular nature of this clump could be demondstrate by racking the objective of the microscope up and down, but cannot adequately be shown in a photograph.

This type of appearance, though commonly seen in carcinomatous pleural effusions or ascitic fluids, is very rare in sputum. × 400.

1 2 3 4

Philps.

It has been stated in the previous paper that vacuolation of the cytoplasm can occur in single non-malignant cells, but in my experience, vacuolation in the cells composing a fragment of tissue is seen only in carcinoma.

*Case 2.*

This case is not so well documented as the previous one. The patient was a woman of 64 who showed miliary shadows in the lung fields.

Clumps of cells, one of which is shown in Fig. 4 were seen in her sputum, which was reported as follows : " Mucoid sputum. Films show clumps of cells which constitute clear evidence of carcinoma. They are of adenocarcinomatous type and do not resemble cells usually seen in primary bronchial carcinomata." An enlarged cervical gland was later removed for histological examination and was reported as showing a mucus-secreting adenocarcinoma, probably of colonic origin. The patient died, and post mortem examination was refused.

It will be noted that the cells shown in the clump in Fig. 4 appear to surround a number of vacuoles, but gross vacuolation is not evident in the cells themselves. The larger mass is hollow, the wall being formed of a single layer of cells.

I make it a practice only to report adenocarcinoma from sputum examination when clumps of the types described are seen. It cannot be too strongly emphasised that single cells containing vacuoles as shown in Fig. 38 of the previous communication do not imply carcinoma.

I am indebted to A. Bligh, A.I.B.P. for the four photographs.

REFERENCE.

PHILPS, F. R. (1954) *Brit. J. Cancer*, **8**, 67.